# Supplementary material for: Time-series transcriptome analysis identified differentially expressed genes in broiler chicken infected with mixed Eimeria species
Source: Front Genet. 2022 Aug 8;13:886781. doi: 10.3389/fgene.2022.886781 (PMC9393255; doi:10.3389/fgene.2022.886781)
Supplement: Supplementary file 2 [file DataSheet1.ZIP › 7dpi_GO.Gsea.1625106495923/GOBP_MICROTUBULE_DEPOLYMERIZATION.html]

Details for gene set GOBP\_MICROTUBULE\_DEPOLYMERIZATION[GSEA]

|  || Dataset | TMM\_7dpi\_gct\_format\_7dpi\_gct\_format.Class\_4dpi.cls #PC\_versus\_NC.Class\_4dpi.cls #PC\_versus\_NC\_repos |
| Phenotype | Class\_4dpi.cls#PC\_versus\_NC\_repos |
| Upregulated in class | 0 |
| GeneSet | GOBP\_MICROTUBULE\_DEPOLYMERIZATION |
| Enrichment Score (ES) | -0.6299839 |
| Normalized Enrichment Score (NES) | -1.9024142 |
| Nominal p-value | 0.0 |
| FDR q-value | 0.06644662 |
| FWER p-Value | 0.404 |
Table: GSEA Results Summary

  

Fig 1: Enrichment plot: GOBP\_MICROTUBULE\_DEPOLYMERIZATION      
 Profile of the Running ES Score & Positions of GeneSet Members on the Rank Ordered List

  

| SYMBOL | TITLE | RANK IN GENE LIST | RANK METRIC SCORE | RUNNING ES | CORE ENRICHMENT || 1 | TRIM54 | na | 1065 | 0.499 | -0.0641 | No |
| 2 | MID1IP1 | na | 1377 | 0.410 | -0.0698 | No |
| 3 | MAP1S | na | 2916 | 0.137 | -0.1913 | No |
| 4 | KATNB1 | na | 4027 | 0.001 | -0.2839 | No |
| 5 | KIF18B | na | 5903 | -0.191 | -0.4309 | No |
| 6 | SPAST | na | 6809 | -0.287 | -0.4922 | No |
| 7 | CLASP2 | na | 8461 | -0.479 | -0.6062 | Yes |
| 8 | KIF2C | na | 8503 | -0.484 | -0.5857 | Yes |
| 9 | KIF2A | na | 8731 | -0.517 | -0.5790 | Yes |
| 10 | TPX2 | na | 8864 | -0.532 | -0.5636 | Yes |
| 11 | CAMSAP1 | na | 9085 | -0.562 | -0.5541 | Yes |
| 12 | TAOK1 | na | 9378 | -0.611 | -0.5481 | Yes |
| 13 | CLASP1 | na | 9464 | -0.626 | -0.5242 | Yes |
| 14 | STMN3 | na | 9624 | -0.649 | -0.5053 | Yes |
| 15 | STMN1 | na | 9701 | -0.661 | -0.4788 | Yes |
| 16 | CAMSAP2 | na | 9813 | -0.676 | -0.4545 | Yes |
| 17 | APC2 | na | 9933 | -0.699 | -0.4298 | Yes |
| 18 | STMN2 | na | 9979 | -0.708 | -0.3985 | Yes |
| 19 | NCKAP5 | na | 10041 | -0.718 | -0.3679 | Yes |
| 20 | ATXN7 | na | 10066 | -0.722 | -0.3341 | Yes |
| 21 | KIF24 | na | 10444 | -0.797 | -0.3260 | Yes |
| 22 | KIF18A | na | 10472 | -0.804 | -0.2884 | Yes |
| 23 | APC | na | 10633 | -0.841 | -0.2600 | Yes |
| 24 | MAP1B | na | 10704 | -0.854 | -0.2235 | Yes |
| 25 | CCSAP | na | 10943 | -0.918 | -0.1978 | Yes |
| 26 | CKAP5 | na | 11027 | -0.945 | -0.1578 | Yes |
| 27 | NAV3 | na | 11326 | -1.048 | -0.1307 | Yes |
| 28 | MAP1A | na | 11871 | -1.442 | -0.1046 | Yes |
| 29 | FGF13 | na | 12005 | -2.341 | 0.0004 | Yes |
Table: GSEA details [plain text format]

  

Fig 2: GOBP\_MICROTUBULE\_DEPOLYMERIZATION      
 Blue-Pink O' Gram in the Space of the Analyzed GeneSet

  

Fig 3: GOBP\_MICROTUBULE\_DEPOLYMERIZATION: Random ES distribution      
 Gene set null distribution of ES for **GOBP\_MICROTUBULE\_DEPOLYMERIZATION**
